# Massive MIMO-Based Distributed Signal Detection in Multi-Antenna Wireless Sensor Networks †

**DOI:** 10.3390/s20072005

**Published:** 2020-04-03

**Authors:** Guofeng Wei, Bangning Zhang, Guoru Ding, Bing Zhao, Yimin Wei, Daoxing Guo

**Affiliations:** College of Communications Engineering, Army Engineering University, Nanjing 210007, China; weiguofengxd@163.com (G.W.); bangning_zhang@sina.com (B.Z.); dr.guoru.ding@ieee.org (G.D.); 18652039567@sina.cn (B.Z.); weiym73@126.com (Y.W.)

**Keywords:** Multi-antenna sensor, distributed signal detection, massive MIMO, wireless sensor network

## Abstract

For massive multiple-input multiple-output (MIMO) distributed wireless sensor networks, this paper investigates the role of multi-antenna sensors in improving network perception performance. First, we construct a distributed multi-antenna sensor network based on massive MIMO. By using the anti-fading characteristics of multi-antennas, it is better to achieve accurate detection than the single-antenna sensor network. Based on this, we derive a closed-loop expression for the detection probability of the best detector. Then, we consider the case that the sensor power resources are limited, and thus we want to use finite power to achieve higher detection probability. For this reason, the power was optimized by the alternating direction method of multipliers (ADMM). Moreover, we also prove that only statistical channel state is needed in large-scale antenna scenarios, which avoid the huge overhead of channel state information. Finally, according to the simulation results, the multi-antenna sensor network has better detection performance than the single-antenna sensor network which demonstrates the improved performance of the proposed schemes and also validates the theoretical findings.

## 1. Introduction

### 1.1. Background and Motivation

Signal detection and parameter estimation in wireless sensor networks (WSNs) have been extensively studied [1,2,3], where event monitoring and spectrum sensing are important applications in wireless sensor networks. In event monitoring and spectrum sensing scenarios, many sensors are deployed within a certain area to monitor certain phenomena and spectrum information [4]. Each sensor makes a local observation within its coverage after limited initial processing, which is transmitted to the fusion center (FC). The FC performs data fusion based on the information fed back by the sensors to obtain a global decision [5].

In parallel, large-scale multiple-input multiple-output (MIMO) technology, also known as massive MIMO, is studied as a basic technology for fifth-generation (5G) wireless communication systems that meet the demand for higher data rate [6,7,8]. In recent years, the application of large-scale MIMO communication technology in wireless sensor networks has attracted wide attention. This is because the large antenna array used by the base station (or convergence center) can simultaneously serve multiple sensors [9], which brings various benefits such as improved spectral efficiency, reduced delay, simplified signal processing [10], improved reliability and power efficiency [11].

However, most existing research efforts consider that the sensor is equipped with a single antenna. As far as we know, multi-antenna technology can effectively resist channel fading, further increase system throughput and meet the needs of transmitting big data [12]. The advantages of multi-antennas in WSN detection problems have recently been studied in [13,14], which inspired us to consider the performance impact of multiple antennas in detection networks. We consider using multi-antenna technology to suppress interference between sensors and FC [15]. In addition, in the evolution of communication antenna technology, multiple antennas will be more applied to the equipment. Therefore, we hold interests in studying the role of multi-antenna sensors in distributed signal detection networks.

### 1.2. Related Work

There have been nearly 40 years of research on distributed sensor detection networks [16], and the research on distributed detection-based massive MIMO networks has been studied in [17,18]. Typical applications include spectrum sensing in cognitive radio networks (CRNs) [19] and event detection in wireless sensor networks (WSNs) [20,21]. With the rapid development of massive MIMO technology, the use of multi-antenna sensors should be more considered in a distributed detection network.

To achieve higher detection performance in a complex environment, the distributed sensor network has been actively researched in recent years. Specifically, in [22], the authors reviewed a variety of distributed detection technologies, focusing on the local optimal distributed detector. The application of distributed detection in multiple access channels was studied in [23]. The detection performance of distributed sensor networks based on local sensor signals was studied in [24]. In [25], the authors discussed the decision fusion problem of wireless sensor networks in fading channels and proposed a new likelihood ratio-based fusion rule, which only requires channel statistics and does not need to rely on instantaneous channels state information (CSI). Chen and Willett considered a general orthogonal channel model between local sensor and FC, where the likelihood ratio test (LRT) method for local sensor decision was studied [26]. In [27], the authors studied the advantages of multi-antenna technology in WSNs. However, they only considered multiple antennas in the fusion center, without considering the role of the terminal multi-antenna.

With the widespread application of massive MIMO technology in 5G communication [28,29], researchers have studied massive MIMO technology in wireless systems. These studies are not only more focused on cellular communications, but also a relatively small number of studies have analyzed the impact of massive MIMO on distributed detection in WSNs. For the massive MIMO wireless sensor network, an optimally distributed detection framework based on the Neyman–Pearson (NP) criterion was proposed in [30], where they only considered multiple antennas in the FC. The distributed detection network composed of many single-antenna sensors and large-scale multi-antenna fusion center was studied in [31], which further considered the impact of transceiver impairments on detection performance. The optimal power allocation problem in wireless sensor distributed detection networks based on MIMO technology was studied in [5]. Moreover, unlike 5G communication, data fusion needs to be considered in the detection network so that the fusion center can make a global decision, i.e., the goal of communication is more focused on increasing the channel transmission throughput, and the signal detection is more focused on increasing the detection probability to make accurate decisions.

Moreover, ADMM is an algorithm that combines the decomposability of double-rising with the superior convergence of multiplication and has been widely used in optimization problems in recent years. ADMM is powerful and efficient in handling the non-convex problem in such large-scale distributed scenarios, for such complicated 5G and beyond systems [29]. In the next-generation wireless network 5G millimeter-wave communication scenario [32], the ADMM algorithm is used to solve the design problem of wideband hybrid pre-coding in multi-beams. For multi-input multiple-output radar systems. Reference [33] studied constant-mode detection with good correlation characteristics. The design of the waveform is to approximate the non-convex problem by the time domain alternating direction multiplier (ADMM) algorithm. Reference [34] studied the channel estimation of mmWave massive MIMO system based on ADMM. The channel estimation problem was defined as the non-convex kernel norm minimization problem under noisy environment, and a new channel estimation method was proposed in conjunction with ADMM. Therefore, we apply the ADMM to our distributed multi-antenna sensor detection network.

### 1.3. Contributions

In this paper, we investigate a massive MIMO-based distributed detection wireless networks with the multi-antenna sensor which the multi-antenna sensor transmits the sensing information more accurately to the fusion center while resisting channel fading. Furthermore, the FC determining the presence or absence of the signal of interest to make a global decision. The main contributions of this paper are summarized as follows:

- We formulate a system model on multi-antenna sensors distributed detection network, which characterizes the influence of multi-antenna sensors on the performance of the distributed detection network.
- We derive an optimal detector with multi-antenna sensor distributed detection network, and a closed-form expression of detection probability. Based on that, we perform the detection probability upper bound performance analysis on the multi-antenna sensor network which lays a theoretical foundation for the subsequent optimization.
- We transform the power-constrained optimization problem into a non-convex fractional programming problem with multiple optimization variables. Subject to the sum of reporting sensor power, the average power allocation algorithm is compared to verify the superiority of our optimization scheme. In addition, we analyzed the trade-off between the number of fusion center antennas and the reported power.

The rest of this paper is organized as follows. In Section 2, the system model and problem formulation are presented. In Section 3, we derive the closed-form expression for the detection probability. We analyze the extreme performance of multi-antenna sensors and propose a power optimization algorithm in Section 4. Simulation results and analysis are provided in Section 5. Finally, we conclude the paper in Section 6.

### 1.4. Notation

For the sake of convenience, the symbols used herein are summarized below. We use lower-case and upper-case bold letters represent vectors and matrices respectively, and transpose and conjugate transpose with ${\left(\cdot \right)}^{T}$ and ${\left(\cdot \right)}^{H}$, respectively. $I_{M}$ to represent the M×M identify matrix, $diag\{d_{1},\ldots ,d_{N}\}$ is the N×N diagonal matrix, and diagonalize it $d_{i}$ for the *i*-th diagonal element, Pr(·) represents the probability, p(·|·) represents a conditional probability density function, the operators E{·}, exp{·} and ||·|| denote the expectation operator, exponential function, Euclidean norm, respectively.

## 2. System Model

**Figure 1:**
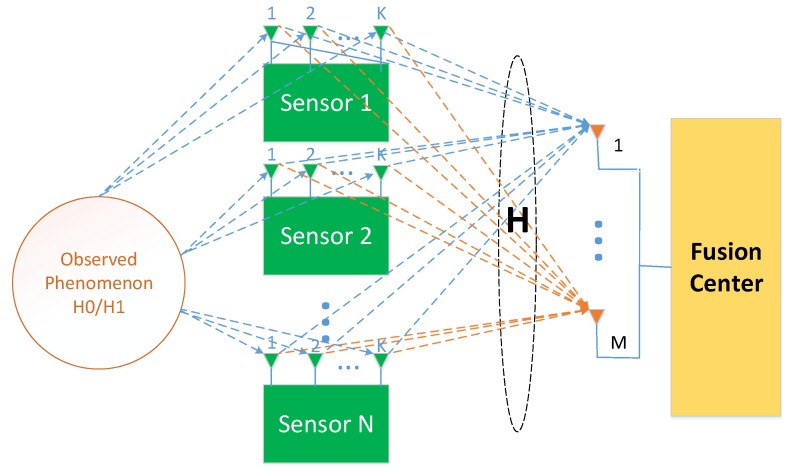
Schematic diagram of massive MIMO-based distributed signal detection in multi-antenna wireless sensor networks.

This paper considers a massive MIMO-based distributed detection in multi-antenna wireless sensor networks, as shown in Figure 1, over a “virtual” multiple-input multiple-output (MIMO) channel between *N* multi-antenna sensors (*K* antennas per sensor) and FC configured with *M* antennas. The sensors will perceive observations of information within their coverage. The local decision on the presence of a signal is sent to the fusion center for global judgment. This scenario can be modeled as a distributed binary hypothesis testing problem [22], where $H_{0}$ and $H_{1}$ indicate the presence and absence of the signal θ, respectively. The sensor measurement model under the two hypotheses is [35]

(1) $$\begin{array}{c} H_{0}\;:\;s_{i}=v_{i}\\ H_{1}\;:\;s_{i}=\theta +v_{i},i=1,\;\ldots ,N, \end{array}$$

where $v_{i}∼CN\left(0,\sigma_{v,i}^{2}\right)$ is the measurement noise of the *i*-th sensor, the signal of interest θ is modeled as a zero-mean circular complex Gaussian variable with variance $\sigma_{\theta }^{2}$, a distribution we denote by $\theta ∼CN(0,\sigma_{\theta }^{2})$.

The *k*-th antenna of the *i*-th sensor amplifies its measurement $s_{i}$ with a gain $g_{ik}$ and forward it to the *M*-antenna FC through a coherent multiple access channel. The received signal at the *m*-th antenna of the FC is

(2) $$\begin{array}{c} y_{m}=\sum_{i=1}^{N}\sum_{k=1}^{K}g_{ik}s_{i}h_{ik,m}+n_{m}, \end{array}$$

where $g_{ik}$ in Equation (2) is the *k*-th antenna gain of the *i*-th sensor, and 1≤i≤N,1≤k≤K,1≤m≤M, $h_{ik,m}$ indicates the channel gain between the *k*-th antenna of the *i*-th sensor and the *m*-th antenna of the FC, and the wireless fading channel is modeled as

(3) $$h_{ik,m}\approx h_{i,m}=\frac{{\tilde{h}}_{i,m}}{\sqrt{d_{i}^{\alpha }}},$$

where $d_{i}$ is the distance between the *i*-th sensor and the FC. Since the different antennas of the same sensor are located similarly, we consider that the distance between the antennas on the same sensor is relatively close, which is far less than the distance between the sensor and the FC, in order to simplify the processing, the channel gain between different antennas of the same sensor and the FC is approximately the same, so $h_{ik,m}\approx h_{i,m}$, α is the path loss index factor, ${\tilde{h}}_{i,m}∼CN\left(0,I_{M}\right)$ is a complex Gaussian vector.

Substituting Equation (2) into Equation (1),

(4) $$\begin{array}{c} \begin{array}{c} H_{0}:y_{m}=\sum_{i=1}^{N}\sum_{k=1}^{K}g_{ik}v_{i}h_{ik,m}+n_{m}\\ H_{1}:y_{m}=\sum_{i=1}^{N}\sum_{k=1}^{K}g_{ik}\theta h_{ik,m}+\sum_{i=1}^{N}\sum_{k=1}^{K}g_{ik}v_{i}h_{ik,m}+n_{m}. \end{array} \end{array}$$

To facilitate derivation and memory, the symbol $g=[g_{11},\ldots ,g_{ik}{]}^{T}$ (dimension: i∗k row, 1 column) is introduced to indicate the antenna gain of each antenna of the sensor. In addition, $g=\sqrt{P}$, $P=[p_{11},\ldots ,p_{ik}]$ (dimension: 1 row, i∗k column) is the reported power of different antennas of *i* sensors, and D$=diag[g_{11},\ldots ,g_{ik}]$ (dimension: i∗k row, i∗k column), $v={\left[v_{11},\ldots ,v_{ik}\right]}^{T}$ (dimension: i∗k row, 1 column) represents the noise on each antenna of the sensor, and H$=[h_{11},\ldots ,h_{\mathrm{ik}}]$ (dimension: *m* row, i∗k column) is the channel gain matrix, where $h_{ik}={\left[h_{ik,1},\ldots ,h_{ik,m}\right]}^{T}$ (dimension: *m* row, 1 column). In distributed multi-antenna wireless sensor networks based on massive MIMO, it is assumed that the matrix H corresponds to the different column vectors for different sensors and is independent of each other, $n={\left[n_{1},\ldots ,n_{M}\right]}^{T}$ (dimension: *m* row, 1 column) $∼CN(0,\sigma_{n}^{2}I_{M})$ is the noise at the fusion center.

Therefore, the above symbols indicate that the signal received at the FC is [36]

(5) $$\begin{array}{c} H_{0}\;:y=\mathrm{HDv}+n\\ H_{1}\;:y=Hg\theta +\mathrm{HDv}+n. \end{array}$$

## 3. Multi-Antenna Sensor Distributed Network Detector

In this section, we determine the presence of the signal based on the received signal y. We distinguish between $D=H_{0}$ and $D=H_{1}$ using a likelihood ratio test (LRT) called the best fusion rule. FC maximizes the probability of detection by a Neyman–Pearson (NP) criterion for a given false alarm probability. The LRT detector works at the threshold γ as follows:

(6) $$L(y)\overset{\Delta }{\;=}\frac{p(y|H_{1})}{p(y|H_{0})}\frac{\overset{H_{1}}{>}}{\underset{H_{0}}{<}}\gamma .$$

In Equation (6), $p(y|H_{1})$ and $p(y|H_{0})$ are the probability density function (PDF) of the receiving matrix is under the alternative hypothesis $H_{1}$ and the null hypothesis $H_{0}$, respectively. According to the Gaussian distribution probability density function $f\left(x\right)=\frac{1}{\sqrt{2\pi \sigma^{2}}}\exp\left(-\frac{{\left(x-\mu \right)}^{2}}{2\sigma^{2}}\right)$, we have [37]

(7) $$\begin{array}{c} p\left(y|H_{0}\right)=p(y_{1},y_{2},\ldots ,y_{j}|H_{0})\\ =\prod_{j=1}^{M}p\left(y_{j}|H_{0}\right)\\ =\frac{1}{{\left(2\pi \right)}^{M/2}\det^{1/2}\left(C_{w}\right)}\exp\left(-\frac{1}{2}y^{H}{C_{w}}^{-1}y\right),\\ p\left(y|H_{1}\right)=p(y_{1},y_{2},\ldots ,y_{j}|H_{1})\\ =\prod_{j=1}^{M}p\left(y_{j}|H_{1}\right)\\ =\frac{1}{{\left(2\pi \right)}^{M/2}\det^{1/2}\left(C_{w}+C_{s}\right)}\exp\left(-\frac{1}{2}y^{H}{\left(C_{w}+C_{s}\right)}^{-1}y\right), \end{array}$$

where $C_{w}=HDVD^{H}H^{H}+\sigma_{n}^{2}I_{M}$ is the covariance of y under $H_{0}$, and $C_{s}=\sigma_{\theta }^{2}Hgg^{H}H^{H}$, so $C_{w}+C_{s}$ is the covariance of y under $H_{1}$, where $V=diag[\sigma_{v,11}^{2},\ldots ,\sigma_{v,ik}^{2}]$ is the measurement noise variance diagonal matrix of multi-antenna sensors.

To get the specific expression of the detector, substituting Equation (7) into Equation (6),

(8) $$\begin{array}{c} \frac{p(y|H_{1})}{p(y|H_{0})}=\frac{\frac{1}{{\left(2\pi \right)}^{M/2}\det^{1/2}\left(C_{w}\;+\;C_{s}\right)}\exp\left(-\frac{1}{2}y^{H}{\left(C_{w}+C_{s}\right)}^{-1}y\right)}{\frac{1}{{\left(2\pi \right)}^{M/2}\det^{1/2}\left(C_{w}\right)}\exp\left(-\frac{1}{2}y^{H}{\left(C_{w}\right)}^{-1}y\right)}\frac{>}{<}\gamma , \end{array}$$

then

(9) $$\begin{array}{c} -y^{H}{\left(C_{w}+C_{s}\right)}^{-1}y\frac{>}{<}\ln\left(\gamma \frac{\det(C_{w}\;+\;C_{s})}{\det(C_{w})}\right)-y^{H}{C_{w}}^{-1}y, \end{array}$$

and we have

(10) $$\begin{array}{c} y^{H}\left[C_{w}^{-1}-{\left(C_{w}+C_{s}\right)}^{-1}\right]y\frac{>}{<}\ln\left(\gamma \left(1+\frac{\sigma_{\theta }^{2}\left(gHH^{H}g^{H}\right)}{C_{w}}\right)\right). \end{array}$$

Furthermore, we can get

(11) $$\sigma_{\theta }^{2}|g^{H}H^{H}C_{w}^{-1}y{|}^{2}\frac{>}{<}\hat{\gamma },$$

where the symbol $\hat{\gamma }=(1+\sigma_{\theta }^{2}f(g))ln[\gamma (1+\sigma_{\theta }^{2}f(g))]$ and $f\left(g\right)=g^{H}H^{H}C_{w}^{-1}Hg$.

**Proof.**  See Appendix A. ☐

The detection probability $P_{D,i}$ and the false alarm probability $P_{FA,i}$ of the *i*-th sensor are defined as

(12) $$\begin{array}{c} P_{D,i}=P_{r}\left(s_{i}=\theta |H_{1}\right)\\ P_{FA,i}=P_{r}\left(s_{i}=\theta |H_{0}\right). \end{array}$$

Based on the obtained LRT detector expression, we evaluate the FC’s detection performance by defining and calculating the detection probability $P_{D}$ and the false alarm probability $P_{FA}$. Specifically, the detection probability that a wireless signal exists and is detected, is defined as [18]

(13) $$\begin{array}{c} \begin{array}{c} P_{D}\overset{\Delta }{\;=}P_{r}(\sigma_{\theta }^{2}|g^{H}H^{H}C_{w}^{-1}y{|}^{2}\geq \hat{\gamma }\left|H_{1}\right). \end{array} \end{array}$$

We can rewrite Equation (13) as

(14) $$\begin{array}{c} P_{D}=P_{r}(\sigma_{\theta }^{2}{\tilde{y}}^{H}W\tilde{y}\geq \hat{\gamma }|H_{1}), \end{array}$$

where $\tilde{y}=(C_{w}+C_{s}{)}^{-\frac{1}{2}}y$ and $W=(C_{w}+C_{s}{)}^{\frac{1}{2}}C_{w}^{-1}\mathrm{Hg}g^{H}H^{H}C_{w}^{-1}(C_{w}+C_{s}{)}^{\frac{1}{2}}.$

From Equation (7) we know that $C_{w}+C_{s}$ under $H_{1}$, so $\tilde{y}=(C_{w}+C_{s}{)}^{-\frac{1}{2}}y$ is distributed as $CN(0,I_{M})$. Based on these, we can define the eigendecomposition of W as

(15) $$\begin{array}{c} W=\mathrm{UG}U^{H}, \end{array}$$

where $G=diag\{f\left(g\right)+\sigma_{\theta }^{2}f{\left(g\right)}^{2},0\ldots 0\}$ and Equation (13) becomes

(16) $$\begin{array}{c} P_{D}=Pr(\sigma_{\theta }^{2}{\tilde{y}}^{H}\mathrm{UG}U^{H}\tilde{y}\geq \hat{\gamma }|H_{1})\\ \overset{\left(a\right)}{=}\mathrm{Pr}(\sigma_{\theta }^{2}{\tilde{y}}^{H}G\tilde{y}\geq \hat{\gamma }|H_{1})\\ \overset{\left(b\right)}{=}\exp\left(\frac{\hat{\gamma }}{\sigma_{\theta }^{4}f{\left(g\right)}^{2}+\sigma_{\theta }^{2}f\left(g\right)}\right), \end{array}$$

where (a) is that the unitary transformation U does not change the distribution of $\tilde{y}$, (b) holds since ${\tilde{y}}^{H}G\tilde{y}$ has a scaled Chi-square distribution with two degrees of freedom.

Similarly, the probability that a wireless signal does not exist and is detected $P_{FA}$ is

(17) $$\begin{array}{c} P_{FA}\overset{\Delta }{\;=}P_{r}(\sigma_{\theta }^{2}|g^{H}H^{H}C_{w}^{-1}y{|}^{2}\geq \hat{\gamma }\left|H_{0}\right)=\exp(-\frac{\hat{\gamma }}{\sigma_{\theta }^{2}f(g)}). \end{array}$$

According to the Neyman–Pearson rule, by adjusting the decision threshold $\hat{\gamma }$, the detection probability $P_{D}$ is maximized under a given false alarm probability $P_{FA}=\varepsilon$. According to Equation (17), we have $\hat{\gamma }=-\ln\left(\varepsilon \right)\sigma_{\theta }^{2}f\left(g\right)$. Substitute $\hat{\gamma }$ into Equation (16), we have

(18) $$P_{D}=\exp\left(\frac{\ln(\varepsilon )}{\sigma_{\theta }^{2}f\left(g\right)+1}\right).$$

## 4. Performance Analysis and Optimization

### 4.1. Detection Performance Analysis

In the previous section, the probability of detection of multi-antenna sensors network at a given false alarm probability was derived. The main obstacle to increasing the size of the antenna array is how to make the energy consumption low enough. In practical applications, because the sensor is small in size, low in cost, and limited in carrying energy, in order to improve the working time of the sensor network, it is necessary to optimize the reporting power of sensors at different positions.

(19) $$\begin{array}{c} \max_{g}f\left(g\right)\\ s.t.\;g^{H}g=P. \end{array}$$

According to Equation (18), ln(ε)<0 (because 0<ε<1), the e-based exponential function is an increasing function, i.e., under the sum of reported power constraint, in order to maximize the detection probability, our objective function f(g) should be designed and maximized. Due to the limited reported power of the sensor, we must consider power constraints to improve the detection performance of the overall network. However, it is not easy to obtain the best g, because it is a question about nonlinear and non-convex optimization. In addition, obtaining the best detection requires a large number of channel matrix H composed of instantaneous CSI channel state information between the sensor and the fusion center [38,39]. Moreover, when the number of antennas at FC becomes very large, the huge overhead in channel estimation is unacceptable. In the next work, we will show that only statistical CSI is needed when *M* becomes large. For further optimization, assuming *M* becomes infinity under the fading wireless channels in Equation (3). Then, we have transformed and asymptotically analyzed f(g) to get the objective function

(20) $$f\left(g\right)\approx \sum_{i=1}^{N}\sum_{k=1}^{K}\frac{Mg_{ik}^{2}}{Mg_{ik}^{2}\left(\sigma_{v,ik}^{2}\right)+d_{ik}^{\alpha }\sigma_{n}^{2}}.$$

**Proof.**  See Appendix B. ☐

We see that f(g) remains asymptotically unchanged as long as $Mg_{ik}^{2}$ is held constant, and achieve asymptotically constant detection performance if any decrease in sensor transmit power is balanced by a corresponding increase in the number of FC antennas [18].

When M→∞, Equation (20) in the optimization problem can be re-expressed as

(21) $$\begin{array}{c} \max_{g_{ik}^{2}}\sum_{i=1}^{N}\sum_{k=1}^{K}\frac{Mg_{ik}^{2}}{Mg_{ik}^{2}\left(\sigma_{v,ik}^{2}\right)+d_{ik}^{\alpha }\sigma_{n}^{2}}\\ s.t.\sum_{i=1}^{N}\sum_{k=1}^{K}g_{ik}^{2}=P. \end{array}$$

Before solving the optimization problem in Equation (21), we analyze the upper bound of the detection probability $P_{D}$ as follows

(22) $$\begin{array}{c} P_{D}\leq {\left\{1+\sigma_{\theta }^{2}\sum_{i=1}^{N}\sum_{k=1}^{K}{\left(\sigma_{v,ik}^{2}\right)}^{-1}\right\}}^{-1} \end{array}$$

When M→∞, P→∞, Equation (22) shows the upper bound of the detection probability that the LRT detector can achieve when given the false alarm probability $P_{FA}$.

**Proof.**  See Appendix C. ☐

Through the previous derivation, it brings important inspiration to improve detection performance. The system has a limited upper limit of detection performance when the number of antennas is large, and depends on the signal variance $\sigma_{\theta }^{2}$, the variance of the receiver noise in the sensor $\sigma_{v,ik}^{2}$ and the number of sensors *N*. It can be seen that the prerequisite for reaching the upper limit of the detection performance is the infinite *M* and *P*, at which time the distribution of the perceived report power becomes unimportant. However, for the actual system, the values of *M* and *P* cannot be infinite, so optimizing $g_{ik}^{2}$ will bring significant benefits to the performance of Equation (21).

To reduce the complexity of subsequent formula derivation, we use new symbols to define $x_{ik}=g_{ik}^{2},a_{ik}=M\sigma_{v,ik}^{2},b_{ik}=d_{ik}^{\alpha }\sigma_{n}^{2}$. Therefore, Equation (21) is compactly rewritten as

(23) $$\begin{array}{c} \max_{\left\{x_{ik}\right\}}\sum_{i=1}^{N}\sum_{k=1}^{K}\frac{Mx_{ik}}{a_{ik}+b_{ik}}\\ s.t.\sum_{i=1}^{N}\sum_{k=1}^{K}g_{ik}^{2}=P,\\ \;g_{ik}^{2}\geq 0,\forall i=1,\ldots ,N,\forall k=1,\ldots ,K. \end{array}$$

This equality constrained optimization problem as a non-convex programming problem is hard to solve [40], which has been proved to be NP-complete [41]. To find an effective solution for Equation (21), we first define

(24) $$\begin{array}{c} t_{i,k}\overset{\Delta }{\;=}\frac{Mx_{ik}}{a_{ik}x_{ik}+b_{ik}},\forall i=1,\ldots ,N,\forall k=1,\ldots ,K. \end{array}$$

Furthermore, we get the equivalent transformation of Equation (21) is

(25) $$\begin{array}{c} \min_{x_{ik},t_{ik}}-\sum_{i=1}^{N}\sum_{k=1}^{K}t_{ik}\\ s.t.\;\;Mx_{ik}-t_{ik}\left(a_{ik}x_{ik}+b_{ik}\right)=0,\\ \;\sum_{i=1}^{N}\sum_{k=1}^{K}g_{ik}^{2}=P,\\ g_{ik}^{2}\geq 0,t_{ik}\geq 0,\forall i=1,\ldots ,N,\forall k=1,\ldots ,K. \end{array}$$

### 4.2. Lagrangian Function Preprocessing

We find an effective solution to the problem by using the augmented Lagrangian function. We first rewrite Equation (25) according to the partial augmented Lagrangian function as

(26) $$\begin{array}{c} L_{1}\left(\left\{x_{ik}\right\},\left\{t_{ik}\right\},\left\{\lambda_{i}\right\}\right)\\ =-\sum_{i=1}^{N}\sum_{k=1}^{K}t_{ik}+\sum_{i=1}^{N}\sum_{k=1}^{K}\lambda_{ik}\left[Mx_{ik}-t_{ik}\left(a_{ik}x_{ik}+b_{ik}\right)\right]\\ \;+\sum_{i=1}^{N}\sum_{k=1}^{K}\frac{\rho_{ik}}{2}{\left(Mx_{ik}-t_{ik}\left(a_{ik}x_{ik}+b_{ik}\right)\right)}^{2}, \end{array}$$

where $\left\{\lambda_{ik}\right\},\left(i=1,\ldots ,N,k=1,\ldots ,K.\right)$ represents the Lagrange multiplier and $\left\{\rho_{\mathrm{ik}}\right\},\left(i=1,\ldots ,N,k=1,\ldots ,K.\right)$ represents the penalty parameter. We use $\mu_{ik}=\frac{\lambda_{ik}}{\rho_{\mathrm{ik}}},\left(i=1,\ldots ,N,k=1,\ldots ,K.\right)$ as a scale factor to describe the relationship between them, and Equation (26) can be rewritten as a scaled form

(27) $$\begin{array}{c} \begin{array}{c} {\tilde{L}}_{1}\left(\left\{x_{ik}\right\},\left\{t_{ik}\right\},\left\{\mu_{ik}\right\}\right)\\ =-\sum_{i=1}^{N}\sum_{k=1}^{K}t_{ik}+\sum_{i=1}^{N}\sum_{k=1}^{K}\frac{\rho_{ik}}{2}(Mx_{ik}-t_{ik}\left(a_{ik}x_{ik}+b_{ik}\right)+\mu_{ik}{)}^{2}. \end{array} \end{array}$$

After we have made these simplification preparations, we can rewrite Equation (25) as

(28) $$\begin{array}{c} \min_{\left\{x_{ik}\right\},\left\{t_{ik}\right\},\left\{\rho_{ik}\right\},\left\{\mu_{ik}\right\}}-\sum_{i=1}^{N}\sum_{k=1}^{K}t_{ik}\\ +\sum_{i=1}^{N}\sum_{k=1}^{K}\frac{\rho_{ik}}{2}{\left(Mx_{ik}-t_{ik}\left(a_{ik}x_{ik}+b_{ik}\right)+\mu_{ik}\right)}^{2}\\ \;s.t.\sum_{i=1}^{N}\sum_{k=1}^{K}x_{ik}=P,\\ x_{ik}\geq 0,t_{ik}\geq 0,\forall i=1,\ldots ,N,\forall k=1,\ldots ,K. \end{array}$$

### 4.3. Power Optimization Algorithm

For many optimization variables, the conventional convex optimization algorithm is hard to find the optimal solution, and ADMM is an important technology for parallel processing and multivariate optimization, which is especially suitable for solving complex optimization problems [42]. We propose an algorithm using the ADMM to solve our problem below, and latter simulation will prove that this will be a very effective algorithm for Equation (28). The specific operation is that Equation (28) can be solved by successive iterations of the original variable $\left\{x_{ik}\right\}$, $\left\{t_{ik}\right\}$ and the double variable $\left\{\rho_{ik}\right\}$, $\left\{\mu_{ik}\right\}$. For k+1 iterations, the algorithm can be summarized into four steps:

- **Step one**: Update ${\{t}_{ik}^{j+1}\}$, and fixed $\left\{x_{ik}^{j}\right\},\left\{\rho_{ik}^{j}\right\},\left\{\mu_{ik}^{j}\right\}$.
  (29) $$\begin{array}{c} {\{t}_{ik}^{j+1}\}=\arg\min_{t_{ik}}-\sum_{i=1}^{N}\sum_{k=1}^{K}t_{ik}\\ +\sum_{i=1}^{N}\sum_{k=1}^{K}\frac{\rho_{ik}^{j}}{2}{\left(Mx_{ik}^{j}-t_{ik}^{j}\left(a_{ik}x_{ik}^{j}+b_{ik}\right)+\mu_{ik}^{j}\right)}^{2}\\ s.t.\;t_{ik}\geq 0,\forall i=1,\ldots ,N,\forall k=1,\ldots ,K. \end{array}$$
  Obviously, this belongs to a multivariate quadratic programming problem, which is cumbersome to solve, but we find that the variable *t* is not coupled with other variables. Therefore, we can find the vertices of the parabola by decomposing and optimizing the variables in Equation (29), and we get the closed-form expression solution as
  (30) $$\begin{array}{c} \begin{array}{c} {\{t}_{ik}^{j+1}\}=\arg\min_{t_{ik}\geq 0}\frac{\rho_{ik}^{j}}{2}{\left(Mx_{ik}^{j}-t_{ik}^{j}\left(a_{ik}x_{ik}^{j}+b_{ik}\right)+\mu_{ik}^{j}\right)}^{2}-t_{ik}^{j}\\ =\frac{\rho_{ik}^{j}\left(a_{ik}x_{ik}^{j}+b_{ik}\right)Mx_{ik}^{j}+x_{ik}^{j}\left(a_{ik}x_{ik}^{j}+b_{ik}\right)\mu_{ik}^{j}+1}{\rho_{ik}^{j}{\left(a_{ik}x_{ik}^{j}+b_{ik}\right)}^{2}},\\ \forall i=1,\ldots ,N,\forall k=1,\ldots ,K. \end{array} \end{array}$$
- **Step two**: Update ${\{x}_{ik}^{j+1}\}$, and fixed $\left\{t_{ik}^{j+1}\right\},\left\{\rho_{ik}^{j}\right\},\left\{\mu_{ik}^{j}\right\}$.
  (31) $$\begin{array}{c} \begin{array}{c} {\{x}_{ik}^{j+1}\}=\arg\min_{x_{ik}^{}}\sum_{i=1}^{N}\sum_{k=1}^{K}\frac{\rho_{ik}^{j}}{2}{\left(Mx_{ik}^{j}-t_{ik}^{j}\left(a_{ik}x_{ik}^{j}+b_{ik}\right)+\mu_{ik}^{j}\right)}^{2}\\ s.t.\sum_{i=1}^{N}\sum_{k=1}^{K}x_{ik}^{j}=P,\\ x_{ik}^{j}\geq 0,\forall i=1,\ldots ,N,\forall k=1,\ldots ,K. \end{array} \end{array}$$
  To simplify understanding, we introduce the following notation: $e_{ik}=M-t_{ik}^{j+1}a_{ik}$ and $\sum_{i=1}^{N}\sum_{k=1}^{K}e_{ik}x_{ik}\overset{\Delta }{\;=}r_{ik}$. We can get the equivalent transformation of Equation (31) as follows,
  (32) $$\begin{array}{c} \min_{x_{ik}^{},r_{ik}}\sum_{i=1}^{N}\sum_{k=1}^{K}\frac{\rho_{ik}^{j}}{2}{\left(r_{ik}-t_{ik}^{j+1}b_{ik}+\mu_{ik}^{j}\right)}^{2}\\ s.t.\sum_{i=1}^{N}\sum_{k=1}^{K}x_{ik}^{j}=P,\\ \sum_{i=1}^{N}\sum_{k=1}^{K}e_{ik}x_{ik}=r_{ik},\\ x_{ik}^{j}\geq 0,\forall i=1,\ldots ,N,\forall k=1,\ldots ,K. \end{array}$$
  Furthermore, introduce the notations $x={\left[x_{11},\ldots ,x_{ik}\right]}^{T}$, $r={\left[r_{11},\ldots ,r_{ik}\right]}^{T}$, $Q=\mathrm{diag}(\frac{\rho_{11}^{j}}{2},\ldots ,\frac{\rho_{ik}^{j}}{2})$, $q=\{\rho_{ik}^{j}\left(u_{ik}^{j}-t_{ik}^{j+1}b_{ik}\right)\}$, $z=\sum_{i=1}^{N}\sum_{k=1}^{K}\frac{\rho_{ik}^{j}}{2}{\left(\mu_{ik}^{j}-t_{ik}^{j+1}b_{ik}\right)}^{2}$, $E=\{e_{11},\ldots ,e_{ik}\}$, $1={\left[1,\ldots ,1\right]}^{T}$, the optimization problem in Equation (32) can be equivalently expressed using the above symbols:
  (33) $$\begin{array}{c} \min_{x,r}r^{T}Qr+q^{T}r+z\\ s.t.\;1^{T}x=P,\\ \;\;E_{x}=r,\\ \;\;x\geq 0. \end{array}$$
  We can effectively solve the quadratic programming (QP) problem by existing math tools such as CVX package [43] or the MATLAB optimization toolbox [44].
- **Step three**: Update ${\{\mu }_{ik}^{j+1}\}$, and fixed $\left\{t_{ik}^{j+1}\right\}$, $\left\{x_{ik}^{j+1}\right\}$, $\left\{\;\rho_{ik}^{j}\right\}$.
  (34) $$\begin{array}{c} \mu_{ik}^{j+1}=\mu_{ik}^{j}+Mx_{ik}^{j+1}-t_{ik}^{j+1}\left(a_{ik}x_{ik}^{j}+b_{ik}\right),\forall i=1,\ldots ,N,\forall k=1,\ldots ,K. \end{array}$$
- **Step four**: Update $\left\{\;\rho_{ik}^{j+1}\right\}$.
  (35) $$\begin{array}{c} \rho_{ik}^{j+1}=\tau \rho_{ik}^{j},\forall i=1,\ldots ,N,\forall k=1,\ldots ,K, \end{array}$$
  where the value of τ determines whether the penalty function is constant or variable, the initial selection of penalty parameters in [45] suggests the use of parameters (i.e., τ > 1) to improve convergence performance and reduce losses.

Due to the large number of iterations of the algorithm, we must care about the convergence of the Algorithm 1. The timeliness of Algorithm 1 is important for cooperative perception, especially some disaster detection and danger information detection scenarios. There are many results discussed in the literature [42] (see Section 3). The convergence of our algorithm is guaranteed under diverse parameters. We will not reproduce the convergence analysis here and we present simulation results to show the convergence performance of Algorithm 1 in Figure 7.
**Algorithm 1** ADMM for Sensor Reporting Power Optimization1:**Input:***N*, *K*, *M*, *P*, $\left\{a_{ik}\right\},\left\{b_{ik}\right\},\tau$.2:**Initialize:** Set $\left\{\rho_{ik}^{0}>0\right\}$ at random and $\{\mu_{ik}^{0}=0\}$, ${\{x}_{ik}^{0}=\frac{P}{N*K}\}$.3:**for**j=0,1,…**do**4:   Update ${\{t}_{ik}^{j+1}\}$ via Equation (30).5:   Update ${\{x}_{ik}^{j+1}\}$ via Equation (31).6:   Update $\left\{\mu_{ik}^{j+1}\right\}$ via Equation (34).7:   Update $\left\{\rho_{ik}^{j+1}\right\}$ via Equation (35).8:**end for**9:**Output:** the optimized sensor reporting power ${\{x}_{ik}^{j+1}\}$.

In addition, we give the following stopping criteria: In successive iterations, the fluctuations of the constraints in the objective function and in Equation (28) are small. Specifically, the following conditions are simultaneously established.

(36) $$\begin{array}{c} \frac{|{\tilde{L_{1}}}^{j+1}-{\tilde{L_{1}}}^{j}|}{|{\tilde{L_{1}}}^{j+1}|}≺\varepsilon_{obj},\\ \frac{|\sum_{i=1}^{N}\sum_{k=1}^{K}x_{ik}^{j+1}-\sum_{i=1}^{N}\sum_{k=1}^{K}x_{ik}^{j}|}{|\sum_{i=1}^{N}\sum_{k=1}^{K}x_{ik}^{j+1}|}≺\varepsilon_{con}, \end{array}$$

where $\varepsilon_{obj}$ and $\varepsilon_{con}$ are convergence tolerances, which can be a smaller constant threshold.

For the computational complexity of Algorithm 1, the dominating cost occurs in line 5 to solve the QP problem. According to [46], the best known running time for an interior point method to solve the QP in Equation (33) is O(N×K), each iteration requiring O(1) arithmetic operations on integers each of which has at most O(N×K) digits, which shows that Algorithm 1 is computationally efficient. In addition, because the sensor is simple and energy limited, it is not suitable for complex calculations, so we perform power optimization based on the statistical channel state at the fusion center.

## 5. Simulation Results

In this section, we using computer simulation to verify the correctness and effectiveness of the theoretical results obtained in previous sections, the simulation parameters which main reference [31] were set as follows: First, we consider N=10 sensors are evenly dispersed at a random distance from the *M* antennas of FC. The distances $d_{i}$ were uniformly distributed over [2, 20], in order to compare the effect of multi-antenna sensor and single-antenna sensor on detection performance in the same environment, we weaken the effect of distance on detection performance. For better illustration, a set of uniformly distributed random distance vectors is selected for subsequent simulation. The set of random distance vectors is [17.5024, 3.3541, 19.4117, 6.4284, 5.9324, 14.0173, 8.3763, 8.6594,19.7629, 11.0626]. The sum of the reported power of all the sensors is P=400 mW, the signal variance $\sigma_{\theta }^{2}=1$, and the noise variance $\sigma_{n}^{2}=0.3$. The false alarm probability $P_{FA}\;=\;\varepsilon \;=\;0.05$ is temporarily given, which means that there is 5% probability that the absence of the signal will be falsely reported as the presence of the signal. In addition, the path loss index factor α=2, signal variance $\sigma_{\theta }^{2}=0.05$, the measurement noise $\sigma_{v,ik}^{2}$ is uniformly distributed in the interval [0.25, 0.5] at the *i*-th sensor.

**Figure 2:**
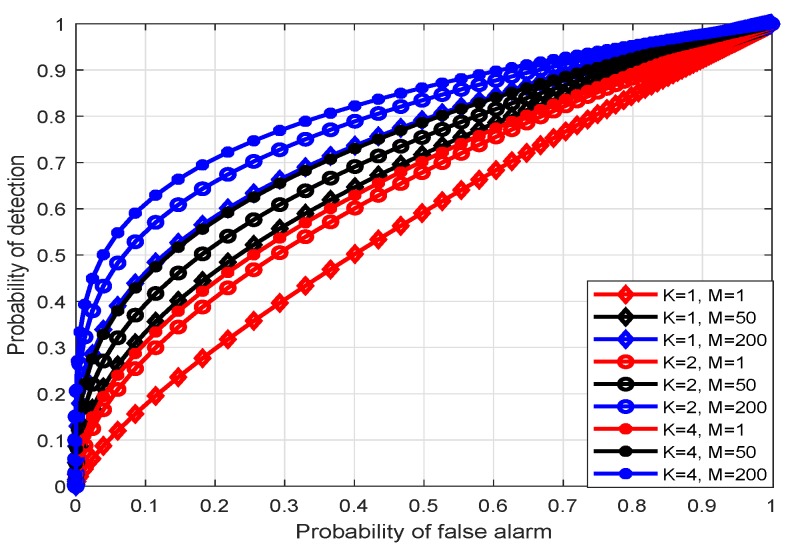
$P_{D}$ vs. $P_{FA}$ when sensor antenna number *K* = 1, 2, 4.

**Figure 3:**
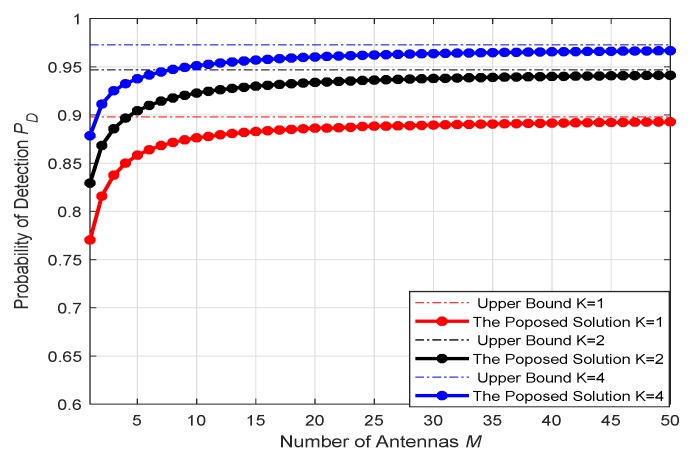
$P_{D}$ vs. *M* when sensor antenna number *K* = 1, 2, 4.

The Figure 2 depicts the relationship between the detection probability ($P_{D}$) and the false positive probability ($P_{FA}$) of the detector when the number of sensor antennas in the multi-antenna sensor network is K=1,2,4. It can be observed that when the number of antennas in the fusion center is fixed, the detection performance of the detector can be improved by using a multi-antenna sensor. When the sensor antenna is determined, the use of different antennas at the FC will also have an impact on the detection performance, with the number of antennas increases, the detection performance will be better, eventually reaching a performance limit as shown in Figure 3, there is still a certain gap between the theoretical value of the upper bound distance, which is caused by noise and actual equipment defects.

**Figure 4:**
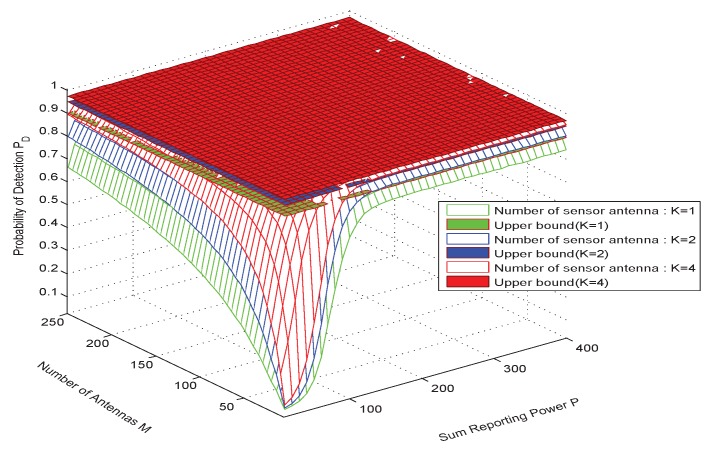
$P_{D}$ vs. *M* and *P*, when sensor antenna number *K* = 1, 2, 4.

In Figure 4, the effect of the number of antennas of the multi-antenna sensor, the number of fusion center antennas and the total reported power on the detection performance are more clearly shown in the multi-antenna sensor network. The simulation results show that the use of multi-antenna sensors will bring better detection performance. We can see from the simulation results that as the number of FC antennas increases, when *M* reaches a certain antenna number (such as 200-250), the detection performance of the proposed solution is very close to the performance ceiling. According to our analysis combined with Equation (22), with the increase of the number of antennas in the fusion center, the reception capability of the fusion center will be stronger, and the detection capability to overcome fading in the transmission channel will be greatly improved, but when it reaches a certain level, the detection performance depends on other factors of the receiver noise, i.e., the certain antenna *M* is dependent on the number of sensor antennas and other variables(e.g., $\sigma_{\theta }^{2}$, $\sigma_{v,ik}^{2}$). In addition, with the infinite increase of the number of fusion center antennas or the unlimited power constraints, the detection performance of the system will reach the upper limit. As deduced from Equation (22), if the number of antennas and power at the FC are infinite, it is not required for power optimization, but in practice, the number of antennas cannot be too large and the power is limited. Therefore, it is important to consider optimizing power. This also gives us a thought that in the use of multi-antenna sensors, in order to obtain good detection performance, we find a trade-off relationship between the number of antennas *M* and the reported total power of *P*. In other words, we can use more antennas at limited power to compensate for the loss performance and vice versa.

**Figure 5:**
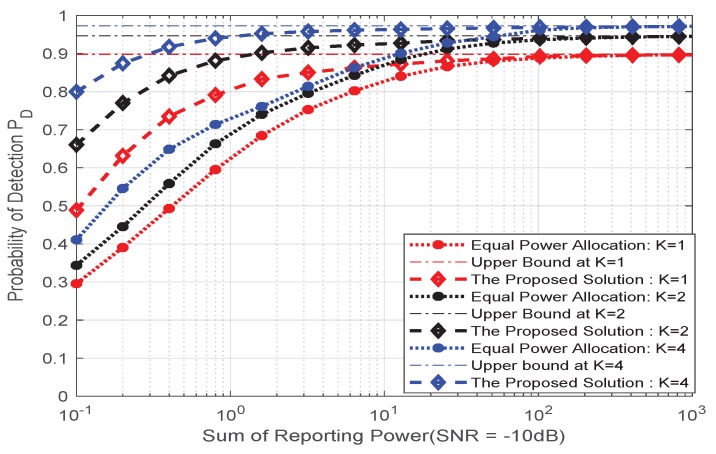
$P_{D}$ vs. *P* for comparison of different methods of detection performance.

Figure 5 depicts the proposed algorithm compared with the average power algorithm at *M* = 250. It can be seen that both algorithms can achieve high detection performance when the reported power *P* is large. When *P* is small through power optimization, our proposed solution can still maintain high detection performance.

**Figure 6:**
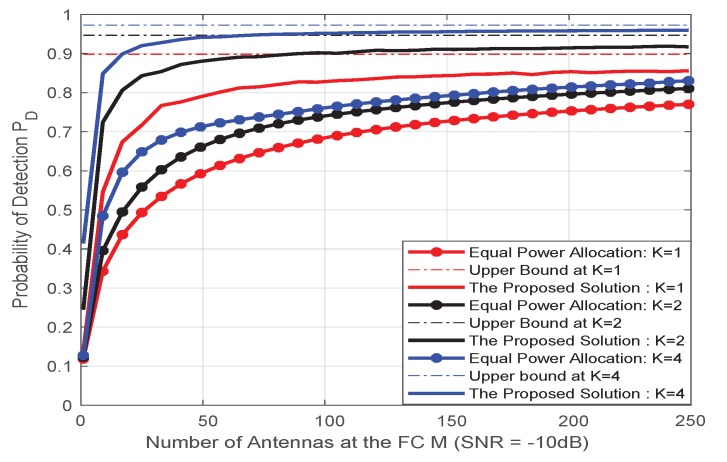
$P_{D}$ vs. *M* for comparison of different methods of detection performance.

Figure 6 shows that the proposed ADMM algorithm compares with the equal power allocation (EPA) algorithm when the power is 400 mW. It can be observed that when the antennas number *M* at FC is large, both ADMM and EPA algorithms can achieve high detection performance, and the proposed algorithm can better approach its performance ceiling. When *M* small, through the power optimization, our proposed solution can still maintain high detection performance. Furthermore, from the power allocation results of different sensors and antennas under the sum of reporting power in Table 1, our algorithm has different power due to different sensor positions and different noise impacts compared to the equal power allocation algorithm. Such as sensor 2, with the number of antennas increases, the required power is smaller. In addition, we can see that when the number of sensor antennas is fixed, the power allocation results of the different sensor antennas are almost the same. According to the analysis, because different antennas of the same sensor are almost the same distance from the FC, and the antenna noise has a slight effect on the power allocation result.

**Figure 7:**
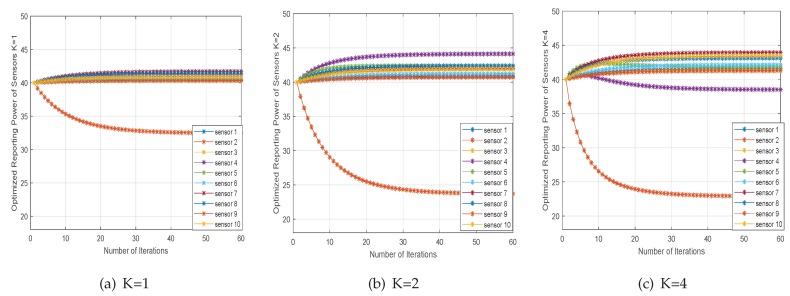
The convergence performance of ADMM.

Figure 7 shows that the convergence performance of the ADMM iterative algorithm using different antenna sensors. Also, we can see that the proposed algorithm reaches a relatively stable state after 30 iterations. In that, a few tens of iterations will produce acceptable results of practical use. The timeliness of the algorithm is important for cooperative perception. This simulation result further validates the convergence speed of the proposed algorithm. For large distributed sensor networks, the faster the convergence speed of the algorithm, the faster the detection speed, which can meet the needs of quick detection.

## 6. Conclusions

In the context of the era of Internet of Things, the perception of the environment is particularly important for the use of detection networks. In this paper, we investigate the role of multi-antenna sensors in improving network perception performance. According to Neyman–Pearson criterion and maximum likelihood test, we derive a closed-loop expression for the detection probability of the best detector. To overcome the power limitation of the sensor, the sensors’ power was optimized by the ADMM. Finally, it is verified by simulation results that the multi-antenna sensor has better detection performance than single-antenna sensor. The optimal detection probability can be obtained with the least energy consumption by optimizing the power. It will be interesting and important to investigate the role of multi-antenna diversity in the detection network and the analysis of detection performance under irrational channel conditions and the presence of device hardware impairments in future work.

## Figures and Tables

**Table 1:** The power allocation results of different sensors and antennas under the sum of reporting power (mW).

Sensor	Distance	Algorithm	K = 1	K = 2		K = 4			
			Antenna 1	Antenna 1	Antenna 2	Antenna 1	Antenna 2	Antenna 3	Antenna 4
Sensor 1	17.5024	EPA	40	20	20	10	10	10	10
		ADMM	40.4157	20.4033	20.4033	10.3786	10.3785	10.3786	10.3785
Sensor 2	3.3541	EPA	40	20	20	10	10	10	10
		ADMM	32.4391	13.6643	13.6643	5.5409	5.5409	5.5409	5.5409
Sensor 3	19.4117	EPA	40	20	20	10	10	10	10
		ADMM	40.3439	20.3348	20.3348	10.3163	10.3163	10.3163	10.3163
Sensor 4	6.4284	EPA	40	20	20	10	10	10	10
		ADMM	41.2436	20.8386	20.8386	10.1989	10.1989	10.1989	10.1989
Sensor 5	5.9324	EPA	40	20	20	10	10	10	10
		ADMM	40.4732	19.9291	19.9291	9.2390	9.2390	9.2390	9.2390
Sensor 6	14.0173	EPA	40	20	20	10	10	10	10
		ADMM	40.6488	20.6298	20.6301	10.5923	10.5923	10.5923	10.5923
Sensor 7	8.3763	EPA	40	20	20	10	10	10	10
		ADMM	41.7096	21.6313	21.6313	11.4725	11.4725	11.4724	11.4725
Sensor 8	8.6594	EPA	40	20	20	10	10	10	10
		ADMM	41.4424	21.3406	21.3406	11.1425	11.1426	11.1425	11.1425
Sensor 9	19.7629	EPA	40	20	20	10	10	10	10
		ADMM	40.3553	20.3509	20.3509	10.3413	10.3413	10.34136	10.3413
Sensor 10	11.0626	EPA	40	20	20	10	10	10	10
		ADMM	40.9284	20.8770	20.8771	10.7777	10.7776	10.7778	10.7776

## Abbreviations

The following abbreviations are used in this manuscript:

MIMO	Multiple-input multiple-output
ADMM	Alternating direction method of multipliers
WSNs	Wireless sensor networks
FC	fusion center
5G	fifth-generation
CRNs	cognitive radio networks
CSI	channels state information
NP	Neyman–Pearson
EPA	Equal power allocation
QP	Quadratic programming
PDF	probability density function

## Appendix A. Proof of Equation (11)

Because Equation (10) is $y^{H}\left(C_{w}^{-1}-{\left(C_{w}+C_{s}\right)}^{-1}\right)y\frac{>}{<}\ln\left(\gamma \left(1+\frac{\sigma_{\theta }^{2}\left(gHH^{H}g^{H}\right)}{C_{w}}\right)\right)$. And $f\left(g\right)=g^{H}H^{H}C_{w}^{-1}Hg$.

The left side of the equal sign of Equation (10) is

(A1) $$\begin{array}{c} y^{H}\left(C_{w}^{-1}-{\left(C_{w}+C_{s}\right)}^{-1}\right)y\\ =yy^{H}\frac{C_{s}}{C_{w}\left(C_{s}+C_{w}\right)}\\ \overset{*C_{w}^{-2}}{=}yy^{H}\frac{C_{s}C_{w}^{-2}}{1+\frac{C_{s}}{C_{w}}}\\ =\frac{\sigma_{\theta }^{2}}{1+\sigma_{\theta }^{2}f\left(g\right)}C_{w}^{-2}yHgg^{H}H^{H}y^{H}\\ =\frac{\sigma_{\theta }^{2}}{1+\sigma_{\theta }^{2}f\left(g\right)}|g^{H}H^{H}C_{w}^{-1}y{|}^{2}. \end{array}$$

The right side of the equal sign of Equation (10) is

(A2) $$\begin{array}{c} \ln(\gamma \left(1+\frac{\sigma_{\theta }^{2}\left(gHH^{H}g^{H}\right)}{C_{w}}\right))\\ =\ln(\gamma \left(1+\sigma_{\theta }^{2}f\left(g\right)\right)). \end{array}$$

So, we have

(A3) $$\begin{array}{c} \sigma_{\theta }^{2}|g^{H}H^{H}C_{w}^{-1}y{|}^{2}\frac{>}{<}\hat{\gamma }. \end{array}$$

Then in the equation $\hat{\gamma }=(1+\sigma_{\theta }^{2}f(g))ln[\gamma (1+\sigma_{\theta }^{2}f(g))]$, and $f\left(g\right)=g^{H}H^{H}C_{w}^{-1}Hg$.

## Appendix B. Proof of Equation (20)

Because $f\left(g\right)=g^{H}H^{H}C_{w}^{-1}Hg$, where $C_{w}=HDVD^{H}H^{H}+\sigma_{n}^{2}I_{M}$.

So, we have

(A4) $$=\begin{array}{c} {{C_{w}}^{-1}=(HDVD^{H}H^{H}+\sigma_{n}^{2}I_{M})}^{-1}\\ {\left(\sigma_{n}^{2}I_{M}\right)}^{-1}-{\left(\sigma_{n}^{2}I_{M}\right)}^{-1}H\left[{\left(DVD^{H}\right)}^{-1}\right.\\ {\left.+H^{H}{\left(\sigma_{n}^{2}I_{M}\right)}^{-1}H\right]}^{-1}H^{H}{\left(\sigma_{n}^{2}I_{M}\right)}^{-1}. \end{array}$$

For further analysis, we use new symbols

(A5) $$\begin{array}{c} T\overset{\Delta }{\;=}{\left(\sigma_{n}^{2}I_{M}\right)}^{-1}\\ \;=\mathrm{diag}\left\{\frac{1}{\sigma_{n}^{2}},\ldots ,\frac{1}{\sigma_{n}^{2}}\right\}\\ E\overset{\Delta }{\;=}DVD^{H}\\ =diag\left\{g_{11},\ldots ,g_{ik}\right\}*\mathrm{diag}\left\{\sigma_{v,11}^{2},\ldots ,\sigma_{v,ik}^{2}\right\}*diag\left\{g_{11},\ldots ,g_{ik}\right\}\\ =\mathrm{diag}\left\{g_{11}^{2}\sigma_{v,11}^{2},\ldots ,g_{ik}^{2}\sigma_{v,ik}^{2}\right\}. \end{array}$$

Further we can get

(A6) $$\begin{array}{c} C_{w}^{-1}=T-TH{\left[E^{-1}+H^{H}TH\right]}^{-1}H^{H}T. \end{array}$$

Substituting Equation (A6) into f(g)

(A7) $$\begin{array}{c} \begin{array}{c} f\left(g\right)=g^{H}H^{H}C_{w}^{-1}Hg\\ =g^{H}H^{H}[T-TH{\left[E^{-1}+H^{H}TH\right]}^{-1}H^{H}T]Hg\\ =g^{H}H^{H}THg-g^{H}H^{H}TH{\left[E^{-1}+H^{H}TH\right]}^{-1}H^{H}THg. \end{array} \end{array}$$

According to the large-scale MIMO theory, as the number of antennas *M* tends to infinity, the random channel vectors between the multi-antenna sensor and the FC gradually become orthogonal, thus we have

(A8) $$\begin{array}{c} \lim_{M\to \infty }\frac{1}{M}H^{H}TH=diag\{\frac{1}{d_{11}^{\alpha }\sigma_{n}^{2}},\ldots ,\frac{1}{d_{ik}^{\alpha }\sigma_{n}^{2}}\}. \end{array}$$

According to Equation (A8), the first term of Equation (A7) is:

(A9) $$\begin{array}{c} \lim_{M\to \infty }g^{H}H^{H}THg=\sum_{i=1}^{N}\sum_{k=1}^{K}\frac{Mg_{ik}^{2}}{\sigma_{n}^{2}d_{ik}^{\alpha }}. \end{array}$$

Part of the second term of Equation (A7) is:

(A10) $$\begin{array}{c} \begin{array}{c} \lim_{M\to \infty }{\left[E^{-1}+H^{H}TH\right]}^{-1}\\ =\lim_{M\to \infty }{\left[diag\left\{\frac{1}{g_{11}^{2}\sigma_{v,11}^{2}},\ldots ,\frac{1}{g_{ik}^{2}\sigma_{v,ik}^{2}}\right\}+diag\left\{\frac{M}{d_{11}^{\alpha }\sigma_{n}^{2}},\ldots ,\frac{M}{d_{ik}^{\alpha }\sigma_{n}^{2}}\right\}\right]}^{-1}\\ =\lim_{M\to \infty }diag\left\{\frac{g_{11}^{2}\sigma_{v,11}^{2}d_{11}^{\alpha }\sigma_{n}^{2}}{Mg_{11}^{2}\sigma_{v,11}^{2}+d_{11}^{\alpha }\sigma_{n}^{2}},\ldots ,\frac{g_{ik}^{2}\sigma_{v,ik}^{2}d_{ik}^{\alpha }\sigma_{n}^{2}}{Mg_{ik}^{2}\sigma_{v,ik}^{2}+d_{ik}^{\alpha }\sigma_{n}^{2}}\right\}. \end{array} \end{array}$$

So, we have

(A11) $$\begin{array}{c} \lim_{M\to \infty }g^{H}H^{H}TH{\left[E^{-1}+H^{H}TH\right]}^{-1}H^{H}THg\\ =\lim_{M\to \infty }\left[g_{11},\ldots ,g_{ik}\right]diag\left\{\frac{M}{d_{11}^{\alpha }\sigma_{n}^{2}},\ldots ,\frac{M}{d_{ik}^{\alpha }\sigma_{n}^{2}}\right\}\\ {}*diag\{\frac{d_{11}^{2}\sigma_{v,11}^{2}d_{11}^{\alpha }\sigma_{n}^{2}}{Md_{11}^{2}\sigma_{v,11}^{2}+d_{11}^{\alpha }\sigma_{n}^{2}},\ldots ,\frac{g_{ik}^{2}\sigma_{v,ik}^{2}d_{ik}^{\alpha }\sigma_{n}^{2}}{Mg_{ik}^{2}\sigma_{v,ik}^{2}+d_{ik}^{\alpha }\sigma_{n}^{2}}\}\\ {}*diag\left\{\frac{M}{d_{11}^{\alpha }\sigma_{n}^{2}},\ldots ,\frac{M}{d_{ik}^{\alpha }\sigma_{n}^{2}}\right\}{\left[g_{11},\ldots ,g_{ik}\right]}^{H}\\ =\lim_{M\to \infty }\left[g_{11},\ldots ,g_{ik}\right]diag\{\frac{M^{2}g_{11}^{2}\sigma_{v,11}^{2}}{d_{11}^{\alpha }\sigma_{n}^{2}\left[Mg_{11}^{2}\sigma_{v,11}^{2}+d_{11}^{\alpha }\sigma_{n}^{2}\right]},\\ \ldots ,\frac{M^{2}g_{ik}^{2}\sigma_{v,ik}^{2}}{d_{ik}^{\alpha }\sigma_{n}^{2}\left[Mg_{ik}^{2}\sigma_{v,ik}^{2}+d_{ik}^{\alpha }\sigma_{n}^{2}\right]}\}{\left[g_{11},\ldots ,g_{ik}\right]}^{H}\\ =\sum_{i=1}^{N}\sum_{k=1}^{K}\frac{M^{2}g_{ik}^{4}\sigma_{v,ik}^{2}}{d_{ik}^{\alpha }\sigma_{n}^{2}\left[Mg_{ik}^{2}\sigma_{v,ik}^{2}+d_{ik}^{\alpha }\sigma_{n}^{2}\right]}. \end{array}$$

Finally, Equations (A9) and (A11) are substituted into Equation (A7) to obtain Equation (A12).

(A12) $$\begin{array}{c} \begin{array}{c} \lim_{M\to \infty }f\left(g\right)\\ =\lim_{M\to \infty }\left(\sum_{i=1}^{N}\sum_{k=1}^{K}\frac{Mg_{ik}^{2}}{\sigma_{n}^{2}d_{ik}^{\alpha }}-\sum_{i=1}^{N}\sum_{k=1}^{K}\frac{M^{2}g_{ik}^{4}\sigma_{v,ik}^{2}}{d_{ik}^{\alpha }\sigma_{n}^{2}\left[Mg_{ik}^{2}\sigma_{v,ik}^{2}+d_{ik}^{\alpha }\sigma_{n}^{2}\right]}\right)\\ =\lim_{M\to \infty }\sum_{i=1}^{N}\sum_{k=1}^{K}\frac{Mg_{ik}^{2}}{Mg_{ik}^{2}\sigma_{v,ik}^{2}+d_{ik}^{\alpha }\sigma_{n}^{2}}. \end{array} \end{array}$$

Finally,

(A13) $$\begin{array}{c} f\left(g\right)\approx \sum_{i=1}^{N}\sum_{k=1}^{K}\frac{Mg_{ik}^{2}}{Mg_{ik}^{2}\sigma_{v,ik}^{2}+d_{ik}^{\alpha }\sigma_{n}^{2}}. \end{array}$$

## Appendix C. Proof of Equation (22)

It can be seen from Equation (20) that for very large *M*, the upper bound of f(g) is

(A14) $$\begin{array}{c} f\left(g\right)=\sum_{i=1}^{N}\sum_{k=1}^{K}\frac{Mg_{ik}^{2}}{Mg_{ik}^{2}\sigma_{v,ik}^{2}+d_{ik}^{\alpha }\sigma_{n}^{2}}\\ \leq \sum_{i=1}^{N}\sum_{k=1}^{K}\frac{Mg_{ik}^{2}}{Mg_{ik}^{2}\sigma_{v,ik}^{2}}\\ =\sum_{i=1}^{N}\sum_{k=1}^{K}\frac{1}{\sigma_{v,ik}^{2}}. \end{array}$$

Substituting Equation (A14) into Equation (18), the upper bound of $P_{D}$ is

(A15) $$\begin{array}{c} P_{D}=\exp\left(\frac{\ln\varepsilon }{\sigma_{\theta }^{2}f\left(g\right)+1}\right)\leq \varepsilon^{{\left\{1+\sigma_{\theta }^{2}\sum_{i=1}^{N}\sum_{k=1}^{K}{\left(\sigma_{v,ik}^{2}\right)}^{-1}\right\}}^{-1}}. \end{array}$$

This completes the proof.
